# Turning an organic semiconductor into a low-resistance material by ion implantation

**DOI:** 10.1088/1468-6996/16/6/065008

**Published:** 2015-12-18

**Authors:** Beatrice Fraboni, Alessandra Scidà, Piero Cosseddu, Yongqiang Wang, Michael Nastasi, Silvia Milita, Annalisa Bonfiglio

**Affiliations:** 1 Dipartimento di Fisica e Astronomia, Università di Bologna, viale Berti Pichat 6/2, 40127 Bologna, Italy; 2 Dipartimento di Ingegneria Elettrica ed Elettronica, Università di Cagliari, piazza d’Armi, 09123 Cagliari, Italy; 3 CNR-INFM S3, via Campi 213/a, 41100 Modena, Italy; 4 Los Alamos National Laboratory, MS-K771, Los Alamos, NM 87545, USA; 5 Nebraska Center for Energy Sciences Research (NCESR), University of Nebraska-Lincoln, NE 68588, USA; 6 Istituto per la Microelettronica e Microsistemi (IMM) Consiglio Nazionale delle Ricerche (CNR), Bologna, Italy

**Keywords:** ion implantation, organic thin film, thin film transistor

## Abstract

We report on the effects of low energy ion implantation on thin films of pentacene, carried out to investigate the efficacy of this process in the fabrication of organic electronic devices. Two different ions, Ne and N, have been implanted and compared, to assess the effects of different reactivity within the hydrocarbon matrix. Strong modification of the electrical conductivity, stable in time, is observed following ion implantation. This effect is significantly larger for N implants (up to six orders of magnitude), which are shown to introduce stable charged species within the hydrocarbon matrix, not only damage as is the case for Ne implants. Fully operational pentacene thin film transistors have also been implanted and we show how a controlled N ion implantation process can induce stable modifications in the threshold voltage, without affecting the device performance.

## 1. Introduction

The performance of organic electronic devices has steadily improved thanks to the advancement in understanding and controlling the organic material molecular structure and electron transport properties. Yet, a few major issues still need to be addressed, such as achieving efficient charge carrier injection [1]. The presence of Schottky barriers at the electrode/semiconductor interface often hinders ohmic injection; therefore, huge efforts have been made in recent years in order to find efficient routes to achieve effective local doping of organic semiconductors in order to both reduce the injection barriers with the electrodes, and also to improve the intrinsic conductivity of the semiconducting layer [2–8]. The implementation of low contact resistance devices usually relies on interface physics (matching the metal electrode work function to the molecular energy levels of the semiconductors), or on dedicated device architectures [9–13]. The electrical doping of organic films has received little attention with respect to doping processes of inorganic semiconductors, possibly because of the relative ease of stacking electron and hole transport organic layers, and the limited stability of chemically doped organic films [14, 15]. However, electrical doping can be a very attractive means to further improve the efficiency of organic devices, e.g. through the realization of a heavily doped organic interface. Such a layer would create a narrow depletion region through which carriers can tunnel increasing injection, similarly to what occurs in inorganic semiconductors.

Ion implantation is a process often used with this aim in the fabrication of inorganic semiconductor devices, but its application to organic materials requires considerable work and attention, due to the fundamental differences exhibited by these materials. Ion irradiation studies have been carried out in the past on polymers [16–18] and showed how the energy transferred by the ion beam into the polymeric chains induces scission and cross-linking effects that strongly alter the material structural, electrical and mechanical properties. However, only a few studies are available on the effects of ion implantation on small molecule organic semiconductors, which are widely used in organic electronics thanks to their excellent transport performance. In particular, very few studies have been performed on fully operating organic devices [19, 20]. We recently reported on low energy ion implantation (20–65 keV) as a tool to reduce and control the degradation of pentacene organic thin film transistors (OTFTs) induced by the exposure to atmosphere (i.e. oxygen and moisture). The effects induced by environmental exposure have been clearly identified and monitored as a function of time, as severe modifications in carrier mobility and threshold. OTFTs that had been implanted with controlled energies and fluences do not suffer from such random variations, and the mobility and threshold voltage values are quite stable in time. In fact, we observed that, despite the strong molecular structure modifications induced by ion implantation, the damage depth distribution can be finely controlled allowing preservation of the functionality of the device and the stabilization of its major transport parameters, e.g. mobility and threshold voltage, over a long period of time [19, 21].

The results we report here focus on the effects induced by a controlled low-energy implantation of Ne^+^ and N^+^ ions on the electrical resistivity in pentacene thin films (300 nm). We performed our experiments on two terminal devices, for standard *I–V* electrical characterization, and also on OTFTs, in which the pentacene layer acted as the active channel of the device. Our results demonstrate that by selectively choosing the Ne^+^ and N^+^ ion energy and dose, it is possible to locally modify the electrical resistivity of the implanted areas up to 6 orders of magnitude. Quite interestingly, we observed that N^+^ ions are much more effective in reducing the material resistivity. Moreover, when ion implantation was performed on fully operating pentacene OTFTs, we have observed that N implantation is very effective in stably pinning the threshold voltage of the device, shifting its values proportionally to the implant depth. In contrast, no significant effect in this sense was observed using Ne^+^ ions. Depth resolved x-ray photoelectron spectroscopy (XPS) analyses, carried out to understand the microstructural origin of these effects, indicated that stable charged chemical species are formed only following the implantation with N ions, which chemically react with the hydrocarbon matrix. In contrast, no stable novel species are formed following Ne implantation [19]. A careful comparison between the damage profiles induced by the different implantation processes allowed us to distinguish the role played by pure implant damage (Ne) and damage associated with the introduction of stable charged species (N), i.e. to assess the occurrence of an electrical ‘doping’ effect.

## 2. Methods

Pentacene films 300 nm thick were deposited by thermal evaporation on a 500 nm silicon dioxide layer thermally grown on top of a highly conductive silicon substrate. The film thickness was monitored by placing the sample close to a quartz crystal microbalance. Pentacene OTFTs were fabricated on a similar substrate using a bottom gate configuration. In this case the highly conductive silicon substrate acts as the gate electrode, whereas the 500 silicon dioxide layer acts as the gate dielectric. All devices were realized using a bottom contact configuration with gold source and drain electrodes (W = 5 mm, L = 50 *μ*m, with W and L the channel width and length, respectively). Ion implantation was performed on a Varian CF-3000 ion implanter at Ion Beam Materials Laboratory in Los Alamos national Laboratory. Monte Carlo SRIM code [24] was used to estimate ion beam parameters that would produce the required damage thickness and ion profile in the pentacene. The implantation was performed at room temperature with two different ion species (N and Ne) at various fluencies/energies. The beam flux was kept at a fixed value so that the flux effect is not a concern in these sets of experiments. To avoid deleterious effects due to an increase of the pentacene film temperature during ion implantation, the samples were mounted on a large nickel cube (4 × 4 × 4 inches) with active air cooling during the implant. The beam flux was relatively modest for all the implants (<10 *μ*A cm^−2^) so the beam heating was not a significant concern. We directly measured the film temperature during the implantation process via a thermocouple attached to the Ni cube and we did not see a temperature rise over 50 °C for the highest fluence used (1 × 10^17^ cm^−2^). Fourier transform infrared (FTIR) spectroscopy was performed to study the modification of the pentacene film by N^+^ or Ne^+^ ions. In order to enhance the signal-to-noise in the IR data, and therefore enhance signal resolution, multiple internal reflection IR (MIR-IR) (also called attenuated total reflectance or ATR), where the IR beam repeatedly interacts with the sample, was employed. The data were acquired under the maximum resolution of 2 cm^−1^ and each spectrum is the average of 400 scans. X-ray reflectivity (XRR) and x-ray diffraction (XRD) in specular geometry (*θ*-2*θ* scan) were carried out with a diffractometer equipped by a rotating anode source (SmartLab-Rigaku). A focus line x-ray beam (Cu K*α* radiation) was collimated by a parabolic graded multilayer mirror placed in front of the sample and double slits were mounted before the detector to achieve the required angular resolution. Atomic force microscopy (AFM) analyses have been carried out with a NSG10 by ND-MDT microscope. All the electrical characteristics of the fabricated devices were carried out at room temperature in air and in the dark with an Agilent HP 4155 Semiconductor Parameter Analyzer. For all the devices, both mobility and threshold voltage were determined from the transfer characteristics in the saturation regime. Electrical resistivity measurements were carried out at room temperature with a 4-point-probe system on Au patterned contact pads.

## 3. Results and discussion

**Figure 1.**
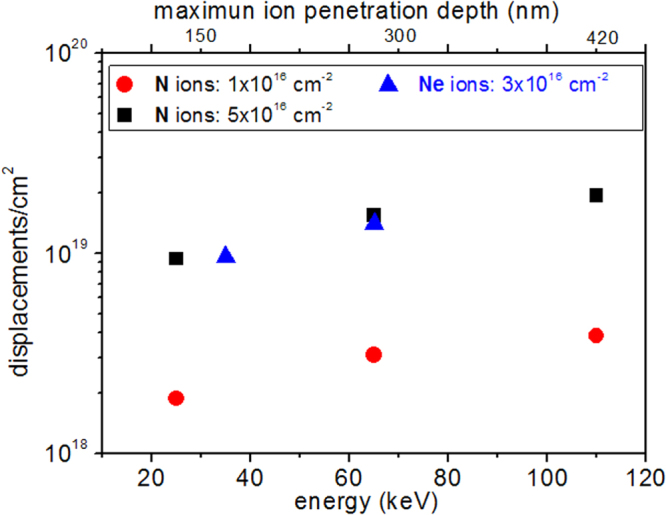
Estimated penetration depth of Ne and N ions, as a function of the implant energy and fluence, evaluated in terms of displacements cm^−2^ by the Monte Carlo SRIM code.

We have implanted 300 nm thick pentacene thin films with different energies (25 to 110 keV) and fluences (1 × 10^14^ to 1 × 10^17^ ions · cm^−2^) to selectively tune the damage entity and depth in the organic film. The penetration depth of the ion beam in the film can be estimated by a Monte Carlo SRIM code [24], and it increases with the implant energy (figure 1). The maximum ion penetration depth is typically defined to be Rp plus ½ of FWHM of the ion depth distribution. The extent of the induced damage, usually measured in number of displacements cm^−2^, varies with the implant fluence. We have chosen to implant with a maximum energy of 110 keV to grant a complete penetration of the 300 nm thick pentacene films studied here. It is noteworthy that comparable damage-depth profiles can be achieved for N and Ne ions for different beam energy and fluences.

**Figure 2.**
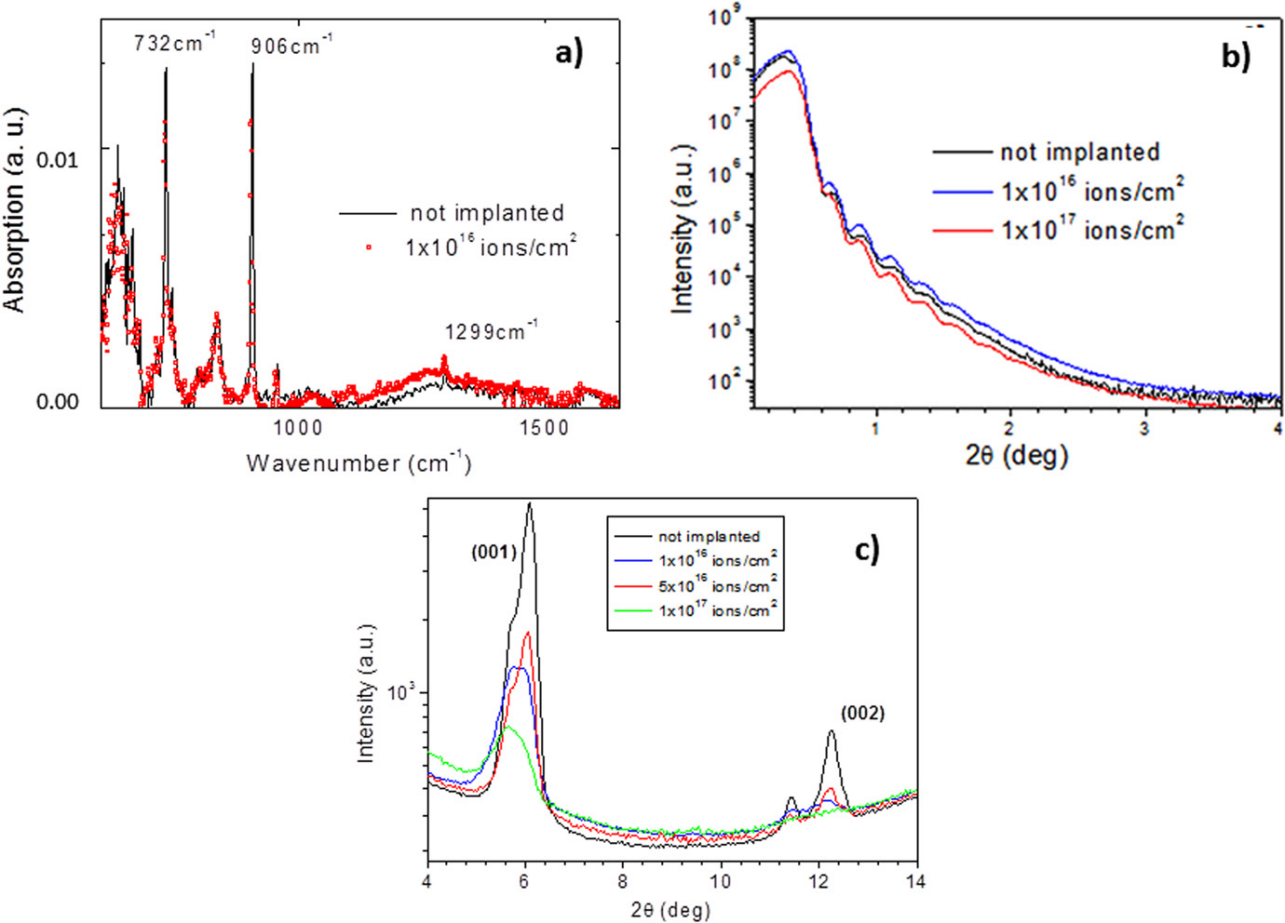
Effects of the N^+^ ion implantation process carried out with different fluences with a beam energy of 25 keV on the pentacene film evaluated by FTIR [19] (a), XRR (b) and XRD (c).

We have studied the ion implantation effects on the hydrocarbon matrix by elastic recoil detection (ERD) and Fourier transform infrared (FTIR) analyses [19] (figure 2(a)). A strong modification is induced in the C–H and C–C molecular bonds (figure 2(a)) forming a hydrogen-depleted carbon-rich matrix. The first two peaks (at 732 and 906 cm^−1^) correspond to a C–H out of plane bending mode, while the peak at 1299 cm^−1^ is associated to a C–C stretching mode. The intensities of the two C–H related peaks decrease after either N^+^ or Ne^+^ implantation while the peak at 1299 cm^−1^ increases. The hydrogen loss increases with the implantation dose [15, 19] without any significant modification of the film thickness as evidenced by the persistence of the same fringe periodicity observed in XRR curves (figure 2(b)). However, a continuous decrease of the film crystallinity is indicated by the progressive weakening of the diffracted peaks (figure 2(c)).

**Figure 3.**
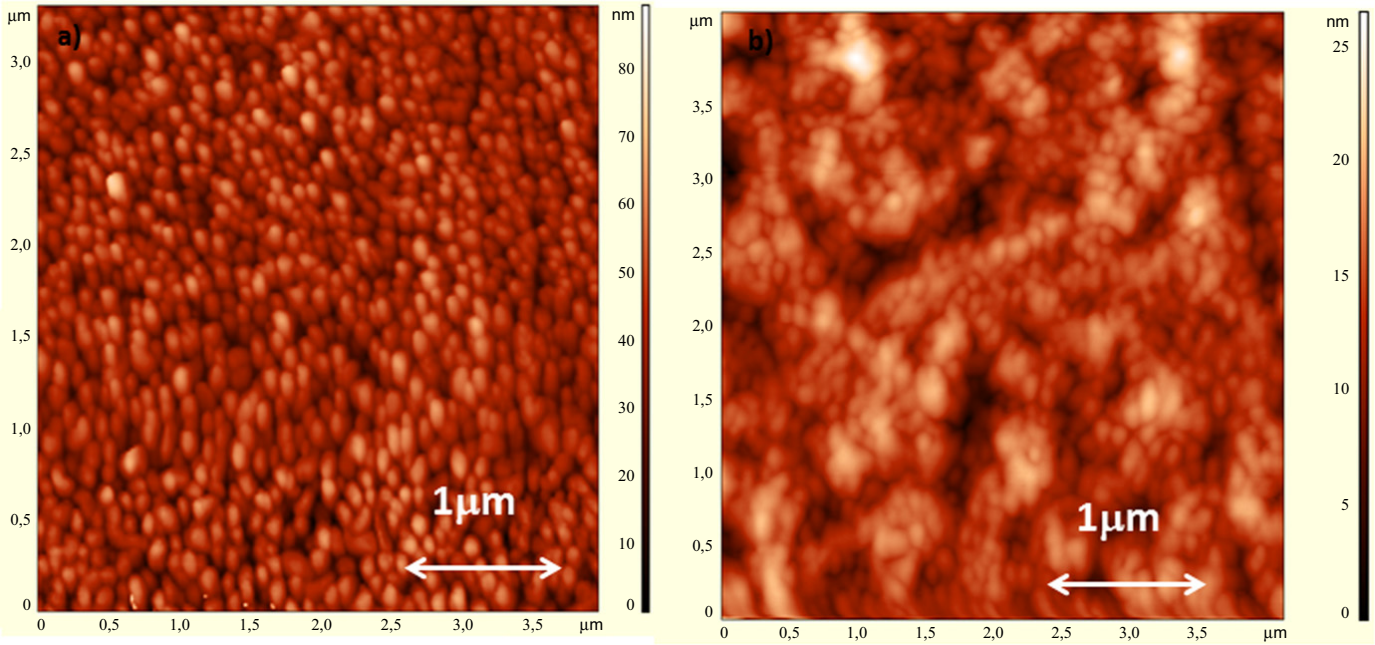
AFM images of pentacene thin films: (a) unimplanted and (b) N^+^ implanted (dose 1 × 10^17^ ions cm^−2^ and energy 55 keV).

AFM analyses have been carried out to investigate how the implantation process affects the film morphology. Figures 3(a) and (b) show the AFM maps of an unimplanted and a N^+^ heavily implanted pentacene thin film and clearly indicate that the implantation process induces the formation of a coarser structure formed by several conglomerates of molecules. This observation is in good agreement with the enhancement of the film conductivity revealed by electrical measurements, as discussed in the following: as the molecules grow closer and more regularly packed, the grain size increases, the grain boundaries fraction decreases and the carrier mean free path and thereby the electrical conductivity of the film increase. This observation agrees with results reported on ion irradiation of thin polymer films [22] and comparable effects are induced by Ne and N ions.

**Figure 4.**
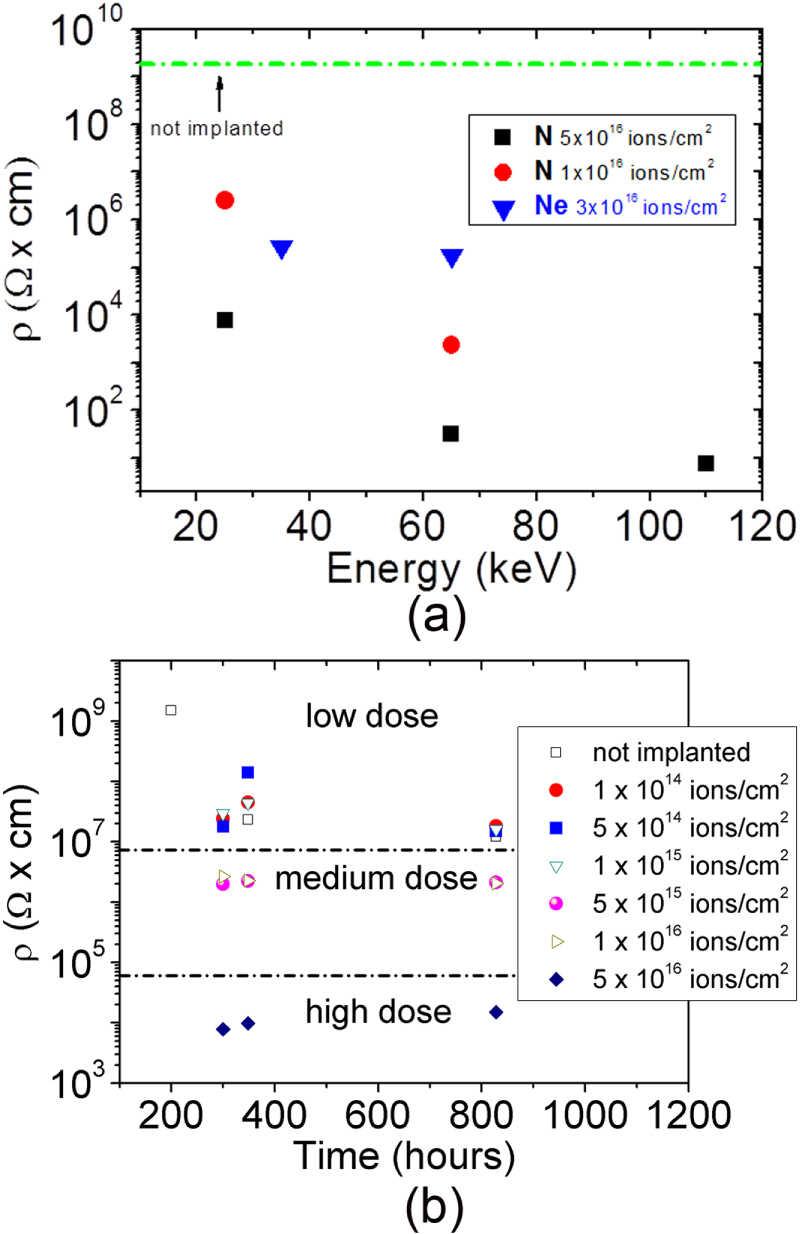
Electrical resistivity of pentacene thin films implanted with different energies and fluences (a) and its evolution in time (b).

Remarkable modifications are induced by Ne and N implantation in the electrical properties of pentacene films. Figure 4 shows the electrical resistivity of films implanted with different energies and fluences. The resistivity of unimplanted samples is about 10^9^ Ω cm and is reported as a dotted line in figure 4(a). Three major comments arise from the data reported in figure 4.

(i) The resistivity of the pentacene film can be varied up to 6 orders of magnitude (compared to the unimplanted film), and the extent of this modification can be tuned by varying the implant fluence and depth.
(ii) The resistivity change is drastically more effective for N implants. In particular, Ne ions (65 keV and 3 × 10^16^ ions · cm^−2^) and N ions (65 keV and 5 × 10^16^ ions · cm^−2^) induce the same amount of damage at the same depth (see figure 1), i.e. over the whole 300 nm film thickness; however, the modification induced in the film resistivity is four orders of magnitude larger for N implants.
(iii) The resistivity modification induced by ion implantation is stable in time, as shown in figure 4(b) for selected N fluences.

The different efficacy of Ne and N implants in modifying the conductivity cannot be ascribed to different macroscopic structural effects (see figure 2), but can be understood thanks to depth-resolved XPS analyses that we have recently reported on implanted pentacene films [19]. No Ne signal was detectable with sputter profile XPS analyses on Ne-implanted samples, indicating (as expected) that implanted Ne did not form bonds with the hydrocarbon matrix, and was thus lost into vacuum prior to, or during, XPS analyses. In contrast, in N-implanted samples we have identified three main N-related chemical states, consistent with –C–N– (398.1 eV), and –C≡N (nitrile functional 399.7 eV) and –NH_3_^+^ functional terminations on the aromatic rings. These observations confirm that N reacts with the hydrocarbon matrix and induces the activation/formation of additional dipole layers (in comparison to unimplanted pentacene) and stable positive charge groups within the pentacene layer.

**Figure 5.**
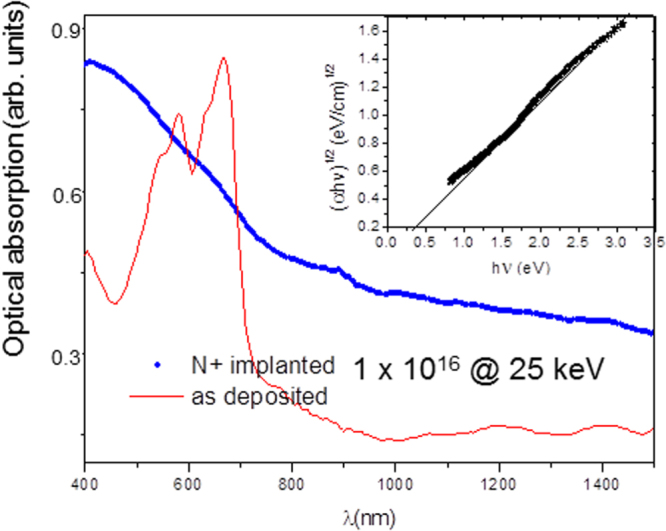
Optical absorption spectra of pentacene thin films as-deposited and after ion implantation with N ions (10^16^ ions cm^−2^ at 25 keV). The inset shows the Tauc plot relative to the implanted sample spectrum.

Indeed, N implantation also induces a strong modification in the density of states distribution, as shown in figure 5, reporting the optical absorption spectra of an unimplanted 300 nm thick pentacene film and of an identical film implanted with a fluence of 1 × 10^16^ ions · cm^−2^ N ions. The observed marked increase in the absorption coefficient can be ascribed to an increased carrier concentration [22]. We can estimate the effective optical band gap of the implanted sample by the Tauc plot [23], shown in the inset of figure 5. The optical band gap of the implanted sample, estimated from the intercept of the curve on the *X* axis, is significantly reduced with respect to virgin pentacene, to a value of about 0.4 eV. This evidence correlates well with the observed increase in the N-implanted material conductivity.

**Figure 6.**
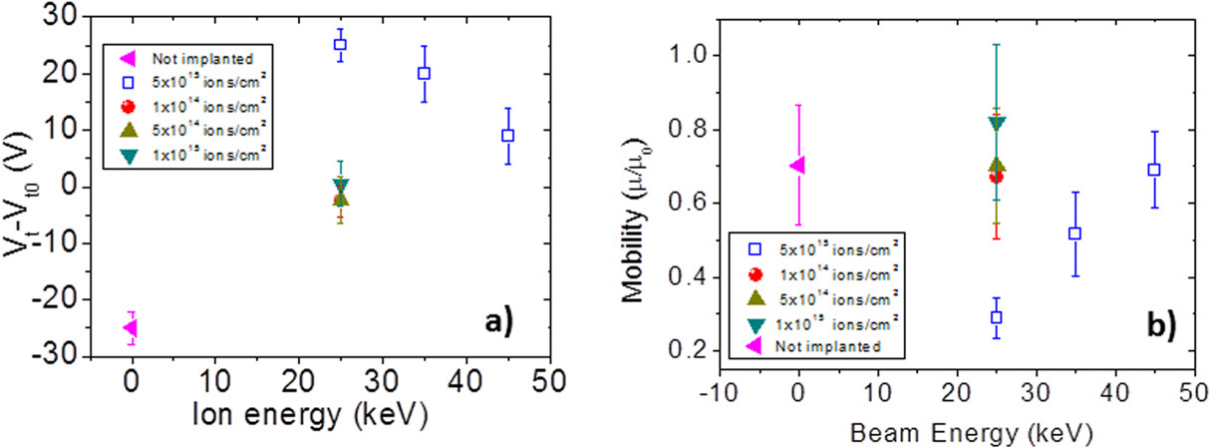
(a) Threshold voltage shift (*V*_t_–*V*_t0_) measured in thin film transistors implanted with different energy and fluence. (b) Mobility variation (*μ*/*μ*_0_) as a function of implant energy and fluence.

We have also implanted and measured fully operational pentacene OTFTs, as we have recently reported how a careful choice of the implant energy and fluence not only allows preservation of the device performance but also reduces its degradation over time [19]. We have observed another very interesting phenomenon on the device threshold voltage, peculiar only to N-implants: implanted OTFTs show a rigid and stable threshold voltage shift that is proportional to the implant energy, i.e. to the thickness of the undamaged layer or to the difference between the film thickness and the implant depth (figure 6). Since our XPS results indicate that N implantation gives rise to a positively charged layer that reaches different depths, controlled by the implant energy, we can hypothesize that the implanted charged layer induces an electric field that superposes to the gate field, thus pushing holes towards the insulating layer, i.e. into the device channel. As a consequence, it is necessary to apply a larger positive gate voltage in order to deplete the channel and to switch the device off.

More interestingly, figure 6(a) shows how low N doses (1 × 10^14^ cm^−2^, 5 × 10^14^ cm^−2^, 1 × 10^15^ cm^−2^) induce a clear stabilization effect on *V*_t_ and, in contrast to what happens to the reference sample and to samples implanted with higher ion doses, *V*_t_ does not differ much from *V*_0_ (the value before implantation). The stabilization effect on *V*_t_, observed also in non *V*_t_-shifted devices, suggests that it is this superposed electric, generated by the N-implanted layer, field (rather than the interface charged states) that determines the effective charge carrier concentration and, as a consequence, the threshold voltage. Figure 6(b) shows how the implantation process does not severely affect the device mobility, in particular for doses lower than 5 × 10^15^ cm^2^ thus, preserving the transistor functionality.

## 4. Conclusions

We have carried out low-energy ion implantation on thin pentacene films with two different types of ions, Ne and N, and we assessed how a strong reduction of the electrical conductivity of the material occurs in both cases, up to eight orders of magnitude with respect to unimplanted pentacene. Moreover, such a reduction is markedly larger for N ions than for Ne ions (provided the same lattice damage is induced), and we ascribed the significantly stronger impact of N ions on the material conductivity to their reactivity within the hydrocarbon matrix, which originates new stable charged species. We could thus assess and identify the role of pure lattice damage (i.e. the one obtained with Ne implantation) and the one of N ion-induced electrically active defects that more effectively intervene on the material transport properties. These observations are in very good agreement with our results on the effects of ion implantation on fully operative pentacene OFETs [15]: implanting N ions can preserve the device functionality and performance and at the same time provides a stable and tunable pinning of the device threshold voltage.

Ion implantation can thus be considered a promising and powerful tool for the permanent modification the electrical transport properties of organic thin films and devices, both in terms of local controlled reduction of the material resistivity (i.e. towards low contact resistance applications) and of the stabilization of major device electrical parameters.
